# Some developments on seasonal INAR processes with application to influenza data

**DOI:** 10.1038/s41598-023-48805-y

**Published:** 2023-12-12

**Authors:** Fatimah E. Almuhayfith, Emmanuel W. Okereke, Manik Awale, Hassan S. Bakouch, Hana N. Alqifari

**Affiliations:** 1 Department of Mathematics and Statistics, College of Science, King Faisal University, Alahsa, 31982 Saudi Arabia; 2 Department of Statistics, Michael Okpara University of Agriculture, Umudike, Nigeria; 3 Department of Statistics, Savitribai Phule Pune University, Pune, 411007 India; 4 Department of Mathematics, College of Science, Qassim University, Buraydah, 51452 Saudi Arabia; 5 Department of Mathematics, Faculty of Science, Tanta University, Tanta, 31111 Egypt; 6 Department of Statistics and Operation Research, College of Science, Qassim University, Buraydah, 51482 Saudi Arabia

**Keywords:** Diseases, Medical research, Mathematics and computing

## Abstract

Influenza epidemic data are seasonal in nature. Zero-inflation, zero-deflation, overdispersion, and underdispersion are frequently seen in such number of cases of disease (count) data. To explain these counts’ features, this paper introduces a flexible model for nonnegative integer-valued time series with a seasonal autoregressive structure. Some probabilistic properties of the model are discussed for general seasonal INAR(p) model and three estimation methods are used to estimate the model parameters for its special case seasonal INAR(1) model. The performance of the estimation procedures has been studied using simulation. The proposed model is applied to analyze weekly influenza data from the Breisgau- Hochschwarzwald county of Baden–Württemberg state, Germany. The empirical findings show that the suggested model performs better than existing models.

## Introduction

Many epidemic diseases follow a seasonal pattern and so is the Influenza. Influenza shows a seasonal pattern^1^ in temperate regions. The weekly/monthly/yearly epidemic data form a time series of counts. Analyzing and forecasting time series of counts remain a useful technique of getting information needed for successful policy making and management of epidemics. Before modeling a count time series, an analyst should be familiar with specific characteristics of the series in order to select a good model for the data. Dispersion characteristic, stationarity, autocorrelation structure and seasonality remain factors that help a researcher to determine the model that should be fitted to a count time series. Seasonality refers to the tendency of a time series (including low count time series) to exhibit patterns or movements that are completed within a year and repeat themselves every year. Seasonality deals with regular and predictable patterns in a time series. For example, a weekly time series of low counts is said to be seasonal with seasonal period s = 52 if its associated autocorrelation function has spikes at multiples of lag 52.

New INAR processes have been constructed and used to model stationary nonseasonal count time series from a variety of fields since the independent works of Al-Osh and Alzaid^2^, and McKenzie^3^, which paved the way for research on the applications and construction of nonnegative integer-valued autoregressive (INAR) processes. Several models were constructed for stationary, overdispersed nonseasonal series based on the binomial thinning operator and first-order autoregressive correlation structure^4,5^. Authors who used other thinning operators to build models for the count time series include Liu and Zhu^6^, and Ristić et al.^7^. There is also evidence of stationary, first-order integer-valued autoregressive processes, which were constructed using innovation distributions, that can exhibit any of the underdispersion, equidispersion and overdispersion phenomena^8–10^.

An INAR(1) process with an innovation distribution that is basically a standard discrete or compound Poisson distribution has been described as inappropriate for modeling any of underdispersed and overdispersed series with either inflation or a deflation of zeros^11^. Zero-modified distributions are useful in modeling count data with an inflation or deflation of zeros. The zero-modified versions of some discrete distributions have been introduced and used as innovation distributions to construct INAR(1) models by some authors. For example, Barreto-Souza^11^ constructed a zero-modified geometric INAR(1) process using the negative binomial thinning operator and illustrated empirically the applicability of the model using two practical data sets. In particular, the model may be used to describe an underdispersed or overdispersed series with deflationary or inflationary zeros. Sharafi et al.^12^ suggested an INAR(1) process using the zero-modified Poisson–Lindley (ZMPL) distributed innovations. They fitted the model to both zero-deflated and zero-inflated series and compared the model’s fit to each data set to that of competing models. Empirically speaking, their model outperformed the other models fitted to each of the data sets.

Sometimes, it is necessary to analyze time series that exhibit seasonal variations. Such time series are called seasonal time series. Seasonal time series of counts are seen in several domains, including epidemiology^13^. Though authors have analyzed several seasonal epidemiological data using the Guassian multiplicative seasonal autoregressive moving average (SARIMA) model^14–16^ and Holt–Winter’s Method^16^, these models may not be suitable for certain nonnegative count time series, especially those involving small counts^17–19^. In the case of seasonal low count time series, a seasonal nonnegative integer-valued autoregressive (INAR) model is needed. Unlike the SARIMA model, the seasonal INAR model takes into consideration the discrete nature of the data through an appropriate discrete innovation distribution. It can also be used to generate integer-valued forecasts. It can also be constructed to account for properties of count time series, such as overdispersion, underdispersion, zero-inflation and zero-deflation. Despite the extensive literature on INAR models, INAR models for stationary seasonal count time series have received little attention^20–22^. Remarkably, none of the seasonal models was developed for the purpose of analyzing an underdispersed or overdispersed seasonal count time series that is either zero-inflated or zero-deflated. This study intends to achieve two principal objectives. First, to propose a seasonal INAR process of order one that is suitable for underdispersed, overdispersed, zero-inflated and zero-deflated count time series data. Second, application of the model to influenza data. In view of the absence of the general seasonal INAR model in the literature, the theoretical framework is developed for the general seasonal INAR model in this paper.

The remainder of this article is organized as follows: “Introduction” discusses the general seasonal INAR model. In “The general seasonal INAR model”, we deal with the construction and properties of the general seasonal INAR(p) model. Methods of estimating the parameters of the model are investigated, namely the Yule–Walker and conditional least squares. The theoretical aspect of forecasting based on the proposed model, simulation and real-world data application for a special case of the general seasonal model, namely the INAR$(1)_{s}$ZMPL process are considered in “Construction of the proposed seasonal INAR(1) process and its properties”. The conclusions of this research are summarized in “Conclusion”.

## The general seasonal INAR model

In response to the comment made by one of the anonymous reviewers, and considering that the literature lacks a general seasonal INAR model, we introduce the general seasonal INAR model in this section. Let “$*$” be the negative binomial thinning (NBT) operator and $\lambda *W= \sum _{i=1}^W Z_i,$ where ${Z_i}$ refers to the sequence of independent and identically distributed (i.i.d.) geometric variables having the probability mass function (PMF)

$$\begin{aligned} P[Z_i=w]=\frac{\lambda ^w}{\left( 1+\lambda \right) ^{w+1}}, w \in \mathbb {N}_0, \lambda \in [0, 1). \end{aligned}$$

An elaborate discussion of the properties of the NBT operator is available in Ristić et al.^7^. The general seasonal INAR model of order *P* and seasonal period *s* (INAR$(P)_s$ model) is defined by

1 $$\begin{aligned} W_t=\lambda _1*W_{t-s}+\lambda _2*W_{t-2s}+\cdots +\lambda _P*W_{t-Ps}+\nu _t, \quad t \in \mathbb {N}_0. \end{aligned}$$

In (1), the counting series are mutually independent, $\lambda _l \in \left[ 0,1\right] ,~l=1, 2, \ldots , P$ and $\left\{ \nu _t \right\}$ are independent of all the counting series.

Theorem 2.1 contains the condition for the existence of a unique stationary INAR$(P)_s$ model.

### Theorem 2.1

*Let* $\left\{ \nu _t \right\}$ *be i.i.d., nonnegative integer-valued random variables whose mean and variance are E*$(\nu _t)= \mu _v$ *and* Var$(\nu _t)= \sigma _\nu ^2$ *respectively. Suppose that* $\lambda _l \in \left[ 0,1\right] ,~l=1, 2, \ldots , P$. *If the roots of the equation*

$$\begin{aligned} z^P-\lambda _1 z^{P-1}-\lambda _2 z^{P-2}-\cdots -\lambda _P=0 \end{aligned}$$

*lie inside the unit circle, then there is a unique stationary nonnegative integer-valued series* $\left\{ W_t \right\}$ *that satisfies the equation*

$$\begin{aligned} W_t=\lambda _1*W_{t-s}+\lambda _2*W_{t-2s}+\cdots +\lambda _P*W_{t-Ps}+\nu _t, \quad t \in \mathbb {N}_0. \end{aligned}$$

### *Proof*

The proof of Theorem 2.1 is based on some properties of the NBT operator. These properties, which were established by Ristić et al.^7^ are as follows

(i) E $$\left[ \prod _{m=1}^r\left( \lambda _m *W_m\right) \right] =\prod _{m=1}^r \lambda _m E\left( W_m\right) , r \ge 1$$
(ii) $$E \left( \lambda *W\right) ^2 =\lambda ^2E \left( W\right) ^2+\lambda (1+\lambda )E \left( W\right)$$
(iii) $E \left( \lambda *W-\lambda *Y \right) ^2=\lambda (1+\lambda )E \left( \vert W-Y \vert \right) +\lambda ^2 E \left( \vert W-Y \vert \right) ^2,$ if each of the counting series of $\lambda *W$ and $\lambda *Y$ has the geometric $\left( \frac{\lambda }{1+\lambda }\right)$ distribution. The remaining part of the proof can be deduced from the proof of Theorem 2.1 in Jin-Guan and Yuan^23^.

$$\square$$

Taking expectation of both sides of (1) and assuming stationarity, the mean of the INAR$(P)_s$ model becomes

2 $$\begin{aligned} \mu _W=E \left( W_t\right) =\frac{\mu _\nu }{1-\sum _{l=1}^P \lambda _l}. \end{aligned}$$

Next, we study the autocorrelation structure of the INAR$(P)_s$ model. Let ${\textbf {W}}_t= \left( W_t, W_{t-s}, W_{t-2s}, \ldots , W_{t-(P+1)s} \right)' .$

Then (1) can be written as

3 $$\begin{aligned} {\textbf {W}}_t={\textbf {C}}*{\textbf {W}}_{t-s}+ \varvec{\nu }_t, \end{aligned}$$

where

$$\begin{aligned} {\textbf {C}}= \begin{pmatrix} \lambda _1&{} \lambda _2&{} \cdots &{} \lambda _{P-1}&{} \lambda _P\\ 1&{}0&{}\cdots &{}0&{}0\\ \vdots &{}\vdots &{}\vdots \vdots \vdots &{}\vdots &{}\vdots \\ 0&{}0&{} \cdots &{}1&{}0 \end{pmatrix} ~\text {and}~ \varvec{\nu }_t= \begin{pmatrix} \nu _t\\ 0\\ \vdots \\ 0 \end{pmatrix}. \end{aligned}$$

The vector of the autocovariances is defined by

$$\begin{aligned} \varvec{\gamma }_k&=E \left[ \left( {\textbf {W}}_t-E({\textbf {W}}_t)\right) \left( {\textbf {W}}_{t-k}-E({\textbf {W}}_{t-k})\right) ^\top \right] \\&=E\left( {\textbf {W}}_t {\textbf {W}}_{t-k}^\top \right) -E\left( {\textbf {W}}_t\right) E\left( {\textbf {W}}_{t-k}^\top \right) \\&={\textbf {C}}E\left( {\textbf {W}}_{t-s} {\textbf {W}}_{t-k}^\top\right) + E\left( \varvec{\nu }_t\right) E\left( {\textbf {W}}_{t-k}^\top \right) -E\left( {\textbf {W}}_t\right) E\left( {\textbf {W}}_{t-k}^\top \right) \\&={\textbf {C}}\varvec{\gamma }_{(k-s)}+\left( {\textbf {C}}-{\textbf {I}}\right) E\left( {\textbf {W}}_{t-s} \right) E\left( {\textbf {W}}_{t-k}^\top \right) +E\left( \varvec{\nu }_t\right) E\left( {\textbf {W}}_{t-k}^\top \right) \end{aligned}$$

Since $E\left( {\textbf {W}}_t\right) ={\textbf {C}}E\left( {\textbf {W}}_{t-s}\right) + E\left( \varvec{\nu }_t\right)$, it is easy to verify that $\left( {\textbf {C}}-{\textbf {I}}\right) E\left( {\textbf {W}}_{t-s} \right) E\left( {\textbf {W}}_{t-k}^\top \right) +E\left( \varvec{\nu }_t\right) E\left( {\textbf {W}}_{t-k}^\top \right)$ is a null matrix. Thus

4 $$\begin{aligned} \varvec{\gamma }_k={\textbf {C}}\varvec{\gamma }_{(k-s)}, ~ k>0. \end{aligned}$$

It follows that the autocovariance function at lag *k* is

5 $$\begin{aligned} \gamma _k=\lambda _1\gamma _{k-s}+\lambda _2\gamma _{k-2s}+\cdots +\lambda _P\gamma _{k-Ps}. \end{aligned}$$

The associated autocorrelation coefficient has the form:

6 $$\begin{aligned} \rho _k=\lambda _1 \rho _{k-s}+\lambda _2 \rho _{k-2s}+\cdots +\lambda _P \rho _{k-Ps}. \end{aligned}$$

In view of (6), it is obvious that the model under consideration and the Gaussian AR model of order *P* and seasonal period *‘s'* have similar autocorrelation structures. Hence, the identification of the model can be done using the autocorrelation function (ACF) and partial autocorrelation function (PACF). Theoretically speaking, the ACF of the INAR$(P)_s$ model has nonzero values at the seasonal lags. The nonzero values at the seasonal lags tail off while the related partial ACF cuts off after lag *P*. When we have a stationary seasonal time series with sample ACF and sample partial ACF that have patterns that are akin to the theoretical ACF and sample partial ACF, we are expected to fit the INAR$(P)_s$ model to the data. In situations where, the sample ACF and sample partial ACF do not look exactly like their theoretical counterparts, a variety of models can be fitted to the given data and the best model is determined using model selection criteria^24^.

The parameters $\lambda _l,~l=1, 2, \ldots , P$ of the model can be estimated using any of the Yule–Walker and conditional least squares approaches. For $k=s, 2s, \ldots , Ps$, we derive *P* equations from (6). These equations are written in matrix form as

$$\begin{aligned} \varvec{\Delta } \varvec{\lambda }= \varvec{\rho }, \end{aligned}$$

where

$$\begin{aligned} \varvec{\Delta }= \begin{pmatrix} 1&{} \rho _s&{} \cdots &{} \rho _{(P-1)s}\\ \rho _s&{}1&{}\cdots &{}\rho _{(P-2)s}\\ \vdots &{}\vdots &{}\vdots \vdots \vdots &{}\vdots \\ \rho _{(P-1)s}&{}\rho _{(P-2)s}&{}\cdots &{}1 \end{pmatrix},~ \varvec{\lambda }= \begin{pmatrix} \lambda _1\\ \lambda _2\\ \vdots \\ \lambda _P \end{pmatrix} ~\text {and}~ \varvec{\rho }= \begin{pmatrix} \rho _s\\ \rho _{2s}\\ \vdots \\ \rho _{Ps} \end{pmatrix}. \end{aligned}$$

The Yule–Walker estimator $\hat{\varvec{\lambda }}$ of $\varvec{\lambda }$ is given as

7 $$\begin{aligned} \hat{ \varvec{\lambda }}= \hat{\varvec{\Delta }} ^{-1}\hat{\varvec{\rho }}. \end{aligned}$$

For example, for a second order monthly count time series, $s=12$ and the estimates $\hat{\lambda }_{1}$ and $\hat{\lambda }_{1}$ based on sample autocorrelation coefficients at lags 12 and 24 are given as.

8 $$\begin{aligned} \begin{pmatrix} \hat{\lambda }_{1}\\ \\ \hat{\lambda }_{1}\\ \end{pmatrix} =\begin{pmatrix} 1 &{} \hat{\rho }_{12}\\ \\ \hat{\rho }_{12}&{} 1\\ \end{pmatrix}^{-1} \begin{pmatrix} \hat{\rho }_{12}\\ \\ \hat{\rho }_{24}\\ \end{pmatrix}. \end{aligned}$$

In (7),

$$\begin{aligned} \hat{\varvec{\Delta }}= \begin{pmatrix} 1&{} \hat{\rho }_s&{} \cdots &{} \hat{\rho }_{(P-1)s}\\ \hat{\rho }_s&{}1&{}\cdots &{}\hat{\rho }_{(P-2)s}\\ \vdots &{}\vdots &{}\vdots \vdots \vdots &{}\vdots \\ \hat{\rho }_{(P-1)s}&{}\hat{\rho }_{(P-2)s}&{}\cdots &{}1 \end{pmatrix},~ \hat{ \varvec{\lambda }}= \begin{pmatrix} \hat{\lambda }_1\\ \hat{\lambda }_2\\ \vdots \\ \hat{\lambda }_P \end{pmatrix} ~\text {and}~ \hat{\varvec{\rho }}= \begin{pmatrix} \hat{\rho }_s\\ \hat{\rho }_{2s}\\ \vdots \\ \hat{\rho }_{Ps} \end{pmatrix}. \end{aligned}$$

Furthermore, the Yule–Walker estimate of $\mu _\nu$ is

9 $$\begin{aligned} \hat{\mu }_\nu =\left( 1-\hat{\lambda }_1-\hat{\lambda }_2-\cdots -\hat{\lambda }_P\right) \bar{W}. \end{aligned}$$

The corresponding estimate of $\sigma _\nu ^2$ is

10 $$\begin{aligned} \hat{\sigma }_\nu ^2=\frac{\sum _{t=Ps+1}^n \left( \hat{\nu }_t-\bar{\nu }_t\right) ^2}{n-Ps}. \end{aligned}$$

Notably,

$$\begin{aligned} \hat{\nu }_t =W_t-\hat{\lambda }_1 W_{t-s}-\hat{\lambda }_2W_{t-2s}-\cdots -\hat{\lambda }_PW_{t-Ps} \end{aligned}$$

and

$$\begin{aligned} \bar{\nu }_t=\frac{\sum _{t=Ps+1}^n \hat{\nu }_t}{n-Ps}. \end{aligned}$$

Let $$\varvec{\Theta }=\left( \mu _\nu , \lambda _1, \lambda _2, \ldots , \lambda _P\right) ^\top$$, $$g(\varvec{\Theta },F_t) =\lambda _1W_{t-s}+\lambda _2W_{t-2s}+\cdots +\lambda _PW_{t-Ps}+\mu _\nu$$ and $$Q(\varvec{\Theta })= \sum _{Ps+1}^n \left( W_t-g(\varvec{\Theta },F_t)\right) ^2.$$

The CLS estimator $\hat{\varvec{\Theta }}$ of $\varvec{\Theta }$ is obtained by minimizing $Q(\varvec{\Theta })$. In this case, we solve the following equations simultaneously:

$$\begin{aligned} \frac{\partial Q(\varvec{\Theta })}{\partial \mu _\nu }&=0;\\ \frac{\partial Q(\varvec{\Theta })}{\partial \lambda _l}&=0,~l=1,2, \ldots ,P. \end{aligned}$$

Certain properties of CLS estimators are well-known (see, Klimko and Nelson^25^).

The minimum mean squared error (MMSE) predictor of $W_{n+1}$ is

$$\begin{aligned} \hat{W}_n(1)&=E\left( W_{n+1} \vert \mathscr {F}_n \right) \\&=\lambda _1W_{n+1-s}+\lambda _2W_{n+1-2s}+\cdots +\lambda _PW_{n+1-Ps}+\mu _\nu \end{aligned}$$

In general, the MMSE of $W_{n+m}$ becomes

11 $$\begin{aligned} \hat{W}_n(m)&=\displaystyle \sum _{l=1}^P E\left( W_{n+m} \vert \mathscr {F}_n \right) \nonumber \\&=\displaystyle \sum _{l=1}^P E\left( \lambda _l*W_{n+m-ls} \vert \mathscr {F}_n \right) +\mu _\nu \nonumber \\&=\displaystyle \sum _{l=1}^P E\left[ E\left( \lambda _l*W_{n+m-ls} \vert W_{n+m-ls}, \mathscr {F}_n \right) \vert \mathscr {F}_n \right] \nonumber \\&=\displaystyle \sum _{l=1}^P \lambda _l E\left( W_{n+m-ls} \vert \mathscr {F}_n \right) \nonumber \\&=\displaystyle \sum _{l=1}^P \lambda _l \hat{W}_n(m-ls)+\mu _\nu . \end{aligned}$$

We have carried out a simulation study to assess the performance of the Yule–Walker (YW) and conditional least squares(CLS) estimates. For the sake of brevity we consider INAR$(2)_{S}$ model and its parameter estimation. We simulated 1000 series from the proposed model for various parameter combinations and for various sample sizes as given in Table 1. We have computed the mean estimates and their related MSEs based on 1000 simulations. In this study, we estimated two autoregressive parameters ($\lambda _{1}$ and $\lambda _{2}$) and the mean of the innovation distribution ($\mu _{\nu }$). For simulation from an innovation distribution, we use the parameter combinations: $\alpha =(4,1,2,0.5)$ and $\delta =(-2,0.5,-1,0.7)$, which give the mean of the innovation distribution as $\mu _{\nu }=(0.9,0.75,1.3333,1.00)$. The estimation of AR coefficient and all the parameters of innovation distribution have been studied in detail for INAR$(1)_{S}$ model in “Construction of the proposed seasonal INAR(1) process and its properties”. From Table 1, it can be seen that the estimates perform well, and their mean squared errors (MSEs) decrease as the sample size increases. It also can be seen that the CLS estimation performs much better than the YW estimation in terms  of the MSE. As the joint distribution of $\lambda _{1} *W_{t-s}$ and $\lambda _{2} *W_{t-2s}$ is not tractable, we can not find the conditional distribution and hence maximum likelihood estimation cannot be attempted, as it has been done for INAR$(1)_{S}$ model in “Construction of the proposed seasonal INAR(1) process and its properties”.

**Table 1:** Parameter estimates and their mean squared errors for INAR$(2)_{S}$ model.

*n*	$\hat{\lambda _1}_{ YW}$	$\hat{\lambda _2}_{ YW}$	$\hat{\mu _{\nu }}_{{ YW}}$	$\hat{\lambda _1}_{{ CLS}}$	$\hat{\lambda _2}_{{ CLS}}$	$\hat{\mu _{\nu }}_{ CLS}$
$\lambda _1=0.2$, $\lambda _2=0.3$, $\mu _{\nu }=0.9$						
300	0.1608	0.1193	1.1578	0.1986	0.2923	0.9120
	0.0055	0.0351	0.0788	0.0081	0.0101	0.0225
500	0.1924	0.1997	1.0531	0.1950	0.2935	0.9154
	0.0025	0.0119	0.0314	0.0031	0.0040	0.0117
1000	0.2035	0.2547	0.9654	0.1989	0.2963	0.9069
	0.0015	0.0033	0.0094	0.0014	0.0015	0.0054
1500	0.2026	0.2711	0.9432	0.1985	0.2974	0.9047
	0.0009	0.0017	0.0054	0.0009	0.0009	0.0035
$\lambda _1=0.3$, $\lambda _2=0.4$, $\mu _{\nu }=0.75$						
300	0.2152	0.1824	1.0753	0.2900	0.3937	0.7690
	0.0116	0.0510	0.1295	0.0073	0.0103	0.0259
500	0.2718	0.2556	0.9927	0.2959	0.3885	0.7670
	0.0038	0.0233	0.0738	0.0032	0.0043	0.0145
1000	0.2978	0.3346	0.8809	0.2969	0.3938	0.7674
	0.0014	0.0055	0.0252	0.0014	0.0016	0.0085
1500	0.3022	0.3563	0.8388	0.2994	0.3962	0.7567
	0.0009	0.0029	0.0129	0.0009	0.0010	0.0050
$\lambda _1=0.4$, $\lambda _2=0.3$, $\mu _{\nu }=1.333$						
300	0.3944	0.1469	1.7433	0.3933	0.2954	1.3582
	0.0110	0.0631	0.4273	0.0090	0.0114	0.0574
500	0.3941	0.1455	1.7445	0.3980	0.2938	1.3547
	0.0027	0.0251	0.1878	0.0036	0.0042	0.0301
1000	0.4165	0.2295	1.5181	0.3990	0.2947	1.3560
	0.0017	0.0062	0.0473	0.0014	0.0015	0.0168
1500	0.4163	0.2525	1.4479	0.3990	0.2973	1.3502
	0.0012	0.0031	0.0232	0.0010	0.0010	0.0124
$\lambda _1=0.6$, $\lambda _2=0.2$, $\mu _{\nu }=1$						
300	0.4066	0.0557	1.5797	0.5912	0.1984	1.0170
	0.0439	0.0244	0.4013	0.0084	0.0097	0.0483
500	0.5138	0.1025	1.4430	0.5915	0.1957	1.0293
	0.0113	0.0119	0.2321	0.0044	0.0043	0.0304
1000	0.5759	0.1510	1.2525	0.5982	0.1965	1.0136
	0.0024	0.0036	0.0806	0.0017	0.0018	0.0159
1500	0.5893	0.1679	1.1633	0.5965	0.1985	1.0156
	0.0012	0.0019	0.0395	0.0012	0.0011	0.0125

## Construction of the proposed seasonal INAR(1) process and its properties

In this section, the definition of the first-order seasonal INAR process based on zero-modified Poisson–Lindley (ZMPL) innovations^12^ and NBT operation is given, with an extensive study. Using the NBT operator, we define the proposed seasonal INAR(1) process as follows.

### Definition 3.1

Let $\lbrace W_t\rbrace$ be a discrete-time nonnegative integer-valued process. Then, the process is a seasonal INAR(1) process with zero-modified Poisson–Lindley innovations if

12 $$\begin{aligned} W_t=\lambda *W_{t-s}+\nu _t, \quad t \in \mathbb {N}_0, \end{aligned}$$

where ‘*s*’ represents the seasonal period such that $s \in \mathbb {N}^+$, $\mathbb {N}^+$ is the set of positive integers, $\lbrace W_t\rbrace$ stands for a sequence of identically distributed nonnegative random variables, $\lbrace \nu _t\rbrace$ is an innovation sequence of i.i.d. ZMPL random variables not depending on the past values of $\lbrace W_t\rbrace$ and the thinnings of $\lbrace W_t\rbrace$ and the random variables $\lbrace W_{t-s}\rbrace$, $\lbrace W_{t-2s}\rbrace$, $\ldots ,$ are independent.

**Figure 1:**
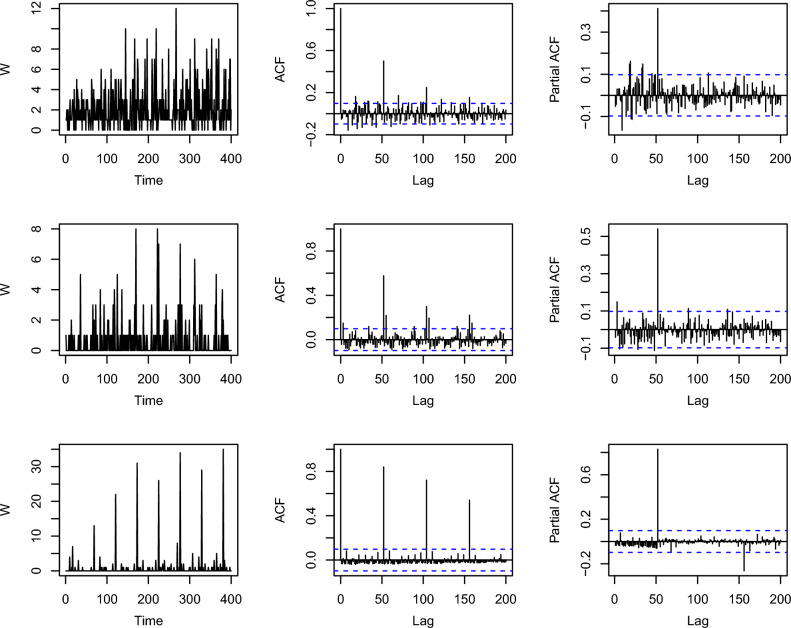
Sample path, sample ACF and sample PACF for simulated data: first row- $\lambda =0.6,~~\alpha =4,~~\delta =-2$; second row- $\lambda =0.7,~~\alpha =5,~~\delta =0$; Third row- $\lambda =0.8,~~\alpha =8,~~\delta =0.5$, seasonal period $S=52$.

In the sequel, the notation INAR(1)$_s$ZMPL process is used to represent the model defined in Eq. (12). A random variable X follows the ZMPL distribution with parameters $\alpha$ and $\delta$ if its PMF is

13 $$\begin{aligned} P(X=x)= {\left\{ \begin{array}{ll} \delta +(1-\delta )\frac{\alpha ^2(\alpha +2)}{(\alpha +1)^3},&{}\quad x=0\\ (1-\delta )\frac{\alpha ^2(x+\alpha +2)}{(\alpha +1)^{x+3}},&{}\quad x \in \mathbb {N^+}, \end{array}\right. } \end{aligned}$$

where $\alpha >0$ and $-\frac{\alpha ^2(\alpha +2)}{\alpha ^2+3\alpha +1} \le \delta \le 1.$ Thus, the unconditional mean and variance of the random variable $\lbrace \nu _t\rbrace$ are, respectively, given by

14 $$\begin{aligned} \text {E}\left( \nu _t\right) =\mu _{\nu }=(1-\delta ) \frac{\alpha +2}{\alpha (\alpha +1)} \end{aligned}$$

and

15 $$\begin{aligned} \text {Var}\left( \nu _t\right) =\sigma ^2_{\nu }=(1-\delta ) \frac{\alpha ^3+4\alpha ^2+6\alpha +2+\delta (\alpha +2)^2}{\alpha ^2 (\alpha +1)^2}. \end{aligned}$$

Following^26^, the distribution of the random variable (RV) $\nu _t$ is overdispersed if $\delta \in [0, 1)$, while it is underdispersed when $\alpha > \sqrt{2}$ and $\delta \in \left( \frac{-\alpha ^2(\alpha +2)}{\alpha ^2+3\alpha +1}, 0 \right) .$ The INAR(1)$_s$ZMPL process comprises ‘*s*’ mutually independent INAR(1) processes with ZMPL innovations, which are constructed using the NBT operator such that they have the autoregressive parameter $\lambda$ and an innovation distribution. The simulated sample paths, sample ACFs and sample PACFs of the process for various parameter combinations are given in the Fig. 1. The seasonality in the simulated data can be seen from the time series plots as well as ACF plots in the figure. Let $W_t^{(r)}=W_{ts+r}, \quad t \in \mathbb {N}_0$, where $\mathbb {N}_0$ is a set of nonnegative integers. For $r=0,1,2, \ldots , s-1$, the process $\lbrace W_t^{(r)} \rbrace$ satisfies the model:

$$\begin{aligned} W_t^{(r)}=\lambda *W_{t-1}^{(r)}+\nu _t^{(r)}, \quad t \in \mathbb {N}_0. \end{aligned}$$

Here, the innovation sequences $\lbrace \nu _t^{(r)} \rbrace$ satisfy the equation $\nu _t^{(r)}=\nu _{ts+r}$. The independence of $\lbrace W_t^{(r)} \rbrace$ is the direct implication of the independence of $\lbrace \nu _t^{(r)} \rbrace$ and the counting processes that define the requisite thinning operators. Additionally, the process defined in Eq. (12) is an *s*-step Markov chain, implying that

$$P\left( W_t=w_t\vert W_{t-1}=w_{t-1}, \ldots , W_0=w_0\right) =P\left( W_t=w_t\vert W_{t-s}=w_{t-s}\right), \quad w_0, w_1, \ldots , w_{s-1} \in \mathbb {N}_0.$$

The conditional mean of the process is

16 $$\begin{aligned} \text {E} \left( W_t \vert W_{t-s} \right)&= \text {E} \left( \lambda *W_{t-s}+\nu _t \vert W_{t-s} \right) \nonumber \\&=\lambda W_{t-s} +\mu _\nu . \end{aligned}$$

Hence, the unconditional expectation of the process is

17 $$\begin{aligned} \text {E} \left( W_t\right)&=\left( \text {E} \left( W_t \vert W_{t-s} \right) \right) \nonumber \\&=\lambda \text {E} \left( W_{t-s}\right) +\mu _\nu . \end{aligned}$$

Assuming stationarity and using Eq. (17), the unconditional mean is found to be

$$\begin{aligned} \text {E} \left( W_t\right) =\frac{\mu _\nu }{1-\lambda }= \mu _W. \end{aligned}$$

The conditional variance satisfies the equation:

18 $$\begin{aligned} \text {Var} \left( W_t \vert W_{t-s} \right)&= \text {Var} \left( \lambda *W_{t-s}+\nu _t \vert W_{t-s} \right) \nonumber \\&=\text {Var} \left( \lambda *W_{t-s}\vert W_{t-s} \right) +\sigma _\nu ^2\nonumber \\&=\lambda ^2\text {E} \left( W_{t-s}^2\vert W_{t-s} \right) +\lambda (\lambda +1)\text {E} \left( W_{t-s}\vert W_{t-s}\right) -\lambda ^2\left( \text {E} \left( W_{t-s}\vert W_{t-s} \right) \right) ^2\nonumber \\&\quad +\sigma _\nu ^2\nonumber \\&=\lambda ^2\text {Var} \left( W_{t-s}\vert W_{t-s} \right) +\lambda (\lambda +1)\text {E} \left( W_{t-s}\vert W_{t-s}\right) +\sigma _\nu ^2\nonumber \\&=\lambda (\lambda +1) W_{t-s}+\sigma _\nu ^2. \end{aligned}$$

The unconditional variance is obtained as

19 $$\begin{aligned} \text {Var} \left( W_t\right)&=\text {E} \left( \text {Var} \left( W_t \vert W_{t-s} \right) \right) +\text {Var} \left( \text {E} \left( W_t \vert W_{t-s} \right) \right) \nonumber \\&=\lambda (\lambda +1) \text {E} \left( W_{t-s}\right) +\sigma _\nu ^2+\lambda ^2 \text {Var} \left( W_{t-s}\right) . \end{aligned}$$

Hence, it follows that

$$\begin{aligned} \text {Var}(W_t)=\frac{\lambda (\lambda +1) \mu _\nu +(1-\lambda )\sigma _\nu ^2}{(1-\lambda )(1-\lambda ^2)}=\sigma _W^2. \end{aligned}$$

Suppose that $W_{(k+h)s+j}$ and $W_{ks+i}$ satisfy the process in Eq. (12), $h \in \mathbb {N}^+$ and $k \in \mathbb {N}_0,~ i, j=1,2, \ldots , s$. In order to gain insight into conditional moments based on the seasonal period as well as the autocorrelation function of the process, we proceed to give the following propositions:

### Proposition 3.1

*The conditional expectation of* $W_{(k+h)s+j}$ *given* $W_{ks+i}$ *is*

$$\begin{aligned} \text {E}\left( W_{(k+h)s+j} \vert W_{ks+i} \right) ={\left\{ \begin{array}{ll} \frac{\mu _\nu }{1-\lambda },&{} \text {if}~i\ne j\\ \lambda ^h W_{ks+i}+(1-\lambda ^h)\mu _W, &{} \text {if}~i= j \end{array}\right. } \end{aligned}$$

*and* $$\lim \nolimits _{h \rightarrow \infty } \text {E}\left( W_{(k+h)s+j} \vert W_{ks+i} \right) =\mu _W.$$

### *Proof*

Let $W_{(k+h)s+j}=W_{k+h}^{(j)}$ and $W_{ks+i}=W_{k}^{(i)}$. If $i\ne j$, the conditional expectation of $W_{k+h}^{(j)}$ given $W_{k}^{(i)}$ is equal to $\frac{\mu _\nu }{1-\lambda }$, which is the unconditional expectation of $W_t$. If $i=j$, we have

$$\begin{aligned} \text {E}\left( W_{(k+h)s+j} \vert W_{ks+i} \right)&= \text {E}\left( W_{k+h}^{(j)} \vert W_{k}^{(j)} \right) \\&=\lambda \text {E}\left( W_{k+h-1}^{(j)} \vert W_{k}^{(j)} \right) + \text {E}(\left( \nu _{k+h}^{(j)} \vert W_{k}^{(i)} \right) \\&=\lambda \text {E}\left( W_{k+h-1}^{(j)} \vert W_{k}^{(j)} \right) +\mu _\nu \\&=\lambda ^2\text {E}\left( W_{k+h-2}^{(j)} \vert W_{k}^{(i)} \right) +(\lambda +1)\mu _\nu \\&.~.~.\\&=\lambda ^h\text {E}\left( W_{k}^{(j)} \vert W_{k}^{(j)} \right) +(\lambda ^{h-1}+\lambda ^{h-2}+\cdots +\lambda +1)\mu _\nu \\&=\lambda ^hW_k^{(j)}+\frac{1-\lambda ^h}{1-\lambda }\mu _\nu \\&=\lambda ^hW_{ks+i}+(1-\lambda ^h)\mu _W. \end{aligned}$$

Consequently,

$$\begin{aligned} \underset{h \rightarrow \infty }{\lim }\ \text {E}\left( W_{(k+h)s+j} \vert W_{ks+i} \right)&= \underset{h \rightarrow \infty }{\lim } \text {E}\left( \lambda ^hW_k^{(j)}+\frac{1-\lambda ^h}{1-\lambda }\mu _\nu \right) \\&=\frac{\mu _\nu }{1-\lambda }\\&=\mu _W. \end{aligned}$$

The proof comes to an end here. $\square$

### Proposition 3.2

*The conditional variance of* $W_{(k+h)s+j}$ *given* $W_{ks+i}$ *is*

$$\begin{aligned} \text {Var}\left( W_{(k+h)s+j} \vert W_{ks+i} \right) ={\left\{ \begin{array}{ll} \frac{\lambda (\lambda +1)\mu _\nu +(1-\lambda )\sigma _\nu ^2}{(1-\lambda )(1-\lambda ^2)},&{} \text {if}~i\ne j\\ (\lambda +1)\left( \frac{\lambda ^{h}(1-\lambda ^h)}{1-\lambda }\right) W_k^{(j)}\\ + \frac{\lambda (\lambda +1)}{1-\lambda } \left( \frac{1-\lambda ^{2(h-1)}}{1-\lambda ^2}-\lambda ^{h-1} \left( \frac{1-\lambda ^{(h-1)}}{1-\lambda }\right) \right) \mu _\nu \\ +\left( \frac{1-\lambda ^{2h}}{1-\lambda ^2}\right) \sigma _\nu ^2, &{} \text {if}~i= j \end{array}\right. } \end{aligned}$$

*and* $$\lim \nolimits _{h \rightarrow \infty } \text {Var}\left( W_{(k+h)s+j} \vert W_{ks+i} \right) =\sigma _W^2.$$

### *Proof*

If $i \ne j$, $W_{k+h}^{(j)}$ and $W_{k}^{(i)}$ are independent. Thus, the conditional variance of $W_{(k+h)s+j}$ given $W_{ks+i}$ is $\frac{\lambda (\lambda +1)\mu _\nu +(1-\lambda )\sigma _\nu ^2}{(1-\lambda )(1-\lambda ^2)}$, the unconditional variance of $W_t$.

If $$i=j$$, then

$$\begin{aligned} \text {Var}\left( W_{(k+h)s+j} \big \vert W_{ks+i} \right)&= \text {Var}\left( W_{k+h}^{(j)} \big \vert W_k^{(j)} \right) \\&=\text {Var}\left( \left( \lambda *W_{k+h-1}^{(j)}+ \nu _{k+h}^{(j)} \right) \big \vert W_k^{(j)} \right) \\&=\text {Var}\left( \lambda *W_{k+h-1}^{(j)}\big \vert W_k^{(j)} \right) +\text {Var}\left( \nu _{k+h}^{(j)} \big \vert W_k^{(j)} \right) \\&=\lambda ^2\text {Var}\left( W_{k+h-1}^{(j)}\big \vert W_k^{(j)} \right) +\lambda (\lambda +1) \text {E}\left( W_{k+h-1}^{(j)}\big \vert W_k^{(j)} \right) \\&\quad +\sigma _\nu ^2\\&=\lambda ^2\text {Var}\left( \lambda *W_{k+h-2}^{(j)}+\nu _{k+h-1}^{(j)}\big \vert W_k^{(j)} \right) \\&\quad +\lambda (\lambda +1) \text {E}\left( W_{k+h-1}^{(j)}\big \vert W_k^{(j)} \right) +\sigma _\nu ^2\\&=\lambda ^4\text {E}\left( (W_{k+h-2}^{(j)})^2\big \vert W_k^{(j)} \right) -\lambda ^4\left( \text {E}\left( W_{k+h-2}^{(j)}\big \vert W_k^{(j)} \right) \right) ^2\\&\quad +\lambda ^3(\lambda +1)\text {E}\left( W_{k+h-2}^{(j)}\big \vert W_k^{(j)} \right) \\&\quad +\lambda (\lambda +1) \text {E}\left( W_{k+h-1}^{(j)}\big \vert W_k^{(j)} \right) +\lambda ^2\sigma _\nu ^2+\sigma _\nu ^2\\&=\lambda ^4\text {Var}\left( W_{k+h-2}^{(j)}\big \vert W_k^{(j)} \right) + \lambda ^3(\lambda +1)\text {E}\left( W_{k+h-2}^{(j)}\big \vert W_k^{(j)} \right) \\&\quad +\lambda (\lambda +1) \text {E}\left( W_{k+h-1}^{(j)}\big \vert W_k^{(j)} \right) +\lambda ^2\sigma _\nu ^2+\sigma _\nu ^2\\&=.~.~.\\&=\lambda ^{2h}\text {Var}\left( W_k^{(j)}\big \vert W_k^{(j)} \right) +\lambda (\lambda +1)M\\&\quad +\left( \lambda ^{2(h-1)}+ \lambda ^{2(h-2)}+\cdots +\lambda ^{2}+1\right) \sigma _\nu ^2, \end{aligned}$$

where

$$\begin{aligned} M&=\lambda ^{2(h-1)}\text {E}\left( W_k^{(j)}\big \vert W_k^{(j)} \right) + \lambda ^{2(h-2)}\text {E}\left( W_{k+1}^{(j)}\big \vert W_k^{(j)} \right) +\cdots + \lambda ^{2}\text {E}\left( W_{k+h-2}^{(j)}\big \vert W_k^{(j)} \right) \\ {}&\quad +\text {E}\left( W_{k+h-1}^{(j)}\big \vert W_k^{(j)} \right) . \end{aligned}$$

Notably, $\text {Var}\left( W_k^{(j)}\big \vert W_k^{(j)} \right) =0$. Also, after some algebra with the earlier discussions, we can find that,

$$\begin{aligned} M&=\frac{\lambda ^{(h-1)}\left( 1-\lambda ^{h}\right) }{1-\lambda }W_{k}^{(j)}+\frac{1}{1-\lambda } \left( \frac{1-\lambda ^{2(h-1)}}{1-\lambda ^{2}}-\frac{\lambda ^{(h-1)}\left( 1-\lambda ^{(h-1)}\right) }{1-\lambda }\right) \mu _\nu . \end{aligned}$$

In the light of the above, we have

$$\begin{aligned} \text {Var}\left( W_{(k+h)s+j} \big \vert W_{ks+i} \right)&= (1+\lambda )\frac{\lambda ^{h}\left( 1-\lambda ^{h}\right) }{1-\lambda }W_{k}^{(j)}\\ {}&\quad +\frac{\lambda (1+\lambda )}{1-\lambda } \left( \frac{1-\lambda ^{2(h-1)}}{1-\lambda ^{2}}-\frac{\lambda ^{(h-1)}\left( 1-\lambda ^{(h-1)}\right) }{1-\lambda }\right) \mu _\nu \\ {}&\quad +\left( \frac{1-\lambda ^{2h}}{1-\lambda ^2}\right) \sigma _\nu ^2.\\ \end{aligned}$$

Hence,

$$\begin{aligned} \underset{h \rightarrow \infty }{\lim }\ \text {Var}\left( W_{(k+h)s+j} \vert W_{ks+i} \right)&=\frac{\lambda (\lambda +1)\mu _\nu +(1-\lambda )\sigma _\nu ^2}{(1-\lambda )(1-\lambda ^2)}=\text {Var}\left( W_t\right) . \end{aligned}$$

$$\square$$

### Proposition 3.3

*The covariance of* $W_{(k+h)s+j}$ *and* $W_{ks+i}$ *has the form*

$$\begin{aligned} \text {Covar}\left( W_{(k+h)s+j},W_{ks+i} \right) ={\left\{ \begin{array}{ll} 0,&{}\text {if}~i\ne j\\ \lambda ^h \frac{\lambda (\lambda +1) \mu _\nu +(1-\lambda )\sigma _\nu ^2}{(1-\lambda )(1-\lambda ^2)},&{}\text {if}~i= j. \end{array}\right. } \end{aligned}$$

### *Proof*

Once $i \ne j$, the variables $W_{(k+h)s+j}$ and $W_{ks+i}$ are uncorrelated. In this case, $\text {Covar}\left( W_{(k+h)s+j}, W_{ks+i} \right) =0.$

$$\mbox{On the other side,}  \begin{aligned} \text {Covar}\left( W_{(k+h)s+j}, W_{ks+i} \right)&= \text {Covar}\left( W_{k+h}^{(j)}, W_{k}^{(j)} \right) \\&=\text {E}\left( (\lambda *W_{k+h-1}^{(j)}+\nu _{k+h}^{(j)} )W_{k}^{(j)} \right) \\&\quad -\text {E}\left( \lambda *W_{k+h-1}^{(j)}+\nu _{k+h}^{(j)}\right) \text {E}\left( W_{k}^{(j)} \right) \\&=\text {E}\left( \left( \displaystyle \sum _{q=1}^{W_{k+h-1}^{(j)}} Z_q\right) W_{k}^{(j)} \right) +\text {E}\left( \nu _{k+h}^{(j)} \right) \text {E}\left( W_{k}^{(j)} \right) \\ {}&\quad -\lambda \text {E}\left( W_{k+h-1}^{(j)}\right) \text {E}\left( W_{k}^{(j)} \right) -\text {E}\left( \nu _{k+h}^{(j)}\right) \text {E}\left( W_{k}^{(j)} \right) \\&=\text {E}\left[ \text {E}\left( \displaystyle \sum _{q=1}^{W_{k+h-1}^{(j)}} Z_q \big \vert W_{k+h-1}^{(j)}\right) \text {E}\left( W_{k}^{(j)} \big \vert W_{k+h-1}^{(j)}\right) \right] \\&\quad -\lambda \text {E}\left( W_{k+h-1}^{(j)}\right) \text {E}\left( W_{k}^{(j)} \right) \\&=\text {E}\left[ {W_{k+h-1}^{(j)}}W_{k}^{(j)} \text {E}\left( Z_q \right) \right] -\lambda \text {E}\left( W_{k+h-1}^{(j)}\right) \text {E}\left( W_{k}^{(j)} \right) \\&=\lambda \text {Cov}\left( {W_{k+h-1}^{(j)}},W_{k}^{(j)} \right) \\&\cdots \\&=\lambda ^h\text {Cov}\left( {W_{k}^{(j)}},W_{k}^{(j)} \right) \\&=\lambda ^h\text {Var}\left( {W_{k}^{(j)}}\right) =\lambda ^h\frac{\lambda (\lambda +1) \mu _\nu +(1-\lambda )\sigma _\nu ^2}{(1-\lambda )(1-\lambda ^2)}. \end{aligned}$$

Dividing the autocovariance function by $\text {Var}\left( {W_t}\right)$, the autocorrelation function (ACF) is obtained as

$$\begin{aligned} \rho (h)= {\left\{ \begin{array}{ll} \lambda ^{\frac{h}{s}},&{}\text { if }h \text { is a multiple of }s\\ 0,&{}\text {elsewhere}. \end{array}\right. } \end{aligned}$$

Here, we have an exponentially decaying autocorrelation function. $\square$

### Techniques for estimating model parameters

Three popular and widely used methods of point estimation for the parameters of the INAR$(1)_{S}$ZMPL are adopted in this section. These are, the Yule–Walker, conditional least squares and conditional maximum likelihood methods. In each of the methods, we assume a realization of the seasonal count time series.

#### Yule–Walker approach

To estimate the parameters $\lambda$, $\delta$ and $\alpha$ of the INAR(1)$_s$ZMPL process, we form three equations by equating $\hat{\rho }(s)$, $\text {E}\left( W_t\right)$ and $\text {Var}\left( W_t\right)$ to $\frac{\hat{\gamma }(s)}{\hat{\gamma }(0)}$, $\overline{W}$ and $\hat{\gamma }(0)$, respectively.

Notably, $\overline{W}=\frac{ \sum _{t=1}^{n} W_t}{n}$, $\hat{\gamma }(s)=\frac{1}{n} \sum _{t=s+1}^{n}\left( W_t-\overline{W}\right) \left( W_{t-s}-\overline{W}\right)$, and *n* are, respectively, the sample mean, sample autocorrelation function and length of the time series. If $\hat{\lambda }_{\text {YW}}$, $\hat{\delta }_{\text {YW}}$ and $\hat{\alpha }_{\text {YW}}$ are the Yule–Walker (YW) estimators of $\lambda$, $\delta$ and $\alpha$ in that order, then the following equations are needed:

20 $$\begin{aligned}&\hat{\lambda }_{YW}=\frac{ \sum _{t=s+1}^{n}\left( W_t-\overline{W}\right) \left( W_{t-s}-\overline{W}\right) }{\sum _{t=1}^{n}\left( W_t-\overline{W}\right) ^2} \end{aligned},$$

21 $$\begin{aligned}&(1-\hat{\delta }_{YW})\frac{\hat{\alpha }_{YW}+2}{\hat{\alpha }_{YW}(\hat{\alpha }_{YW}+1)(1-\hat{\lambda }_{YW})}=\overline{W}\end{aligned},$$

22 $$\mbox{and}~~~~~~~~~~~~~~~~~~~~~~~~~~~~~~~~~~~~~~~~~~~ \begin{aligned}&\frac{A}{(1-\hat{\lambda }_{YW}^2)\hat{\alpha }_{YW}(\hat{\alpha }_{YW}+1)(\hat{\alpha }_{YW}+2)}&=\hat{\gamma }(0), \end{aligned}$$

where

$$\begin{aligned} A&=\hat{\lambda }_{YW}(\hat{\lambda }_{YW}+1)\overline{W}\hat{\alpha }_{YW}(\hat{\alpha }_{YW}+1)(\hat{\alpha }_{YW}+2)\\&\quad +(1-\hat{\lambda }_{YW})\overline{W}\left( (1-(1-\hat{\lambda }_{YW})\overline{W})\hat{\alpha }_{YW}^3 +(5-3(1-\hat{\lambda }_{YW})\overline{W})\hat{\alpha }_{YW}^2\right) \\ {}&\quad +(1-\hat{\lambda }_{YW})\overline{W}\left( (10-2(1-\hat{\lambda }_{YW})\overline{W})\hat{\alpha }_{YW}+6\right) . \end{aligned}$$

From Eq. (20), we obtain

23 $$\begin{aligned} (1-\hat{\delta }_{YW})&=\frac{\hat{\alpha }_{YW}(\hat{\alpha }_{YW}+1)(1-\hat{\lambda }_{YW})}{\hat{\alpha }_{YW}+2}\overline{W}. \end{aligned}$$

Using Eqs. (21) and (22), the following cubic equation is obtained:

24 $$\begin{aligned} c_1\hat{\alpha }_{YW}^3+c_2\hat{\alpha }_{YW}^2+c_3\hat{\alpha }_{YW}+c_4=0, \end{aligned}$$

where

$$\begin{aligned} c_1&=\left[ \hat{\lambda }_{YW}(\hat{\lambda }_{YW}+1)+ (1-\hat{\lambda }_{YW})(1-(1-\hat{\lambda }_{YW})\overline{W})\right] \overline{W}-(1-\hat{\lambda }_{YW}^2)\hat{\gamma }(0),\\ c_2&=\left[ 3\hat{\lambda }_{YW}(\hat{\lambda }_{YW}+1)+ (1-\hat{\lambda }_{YW})(5-3(1-\hat{\lambda }_{YW})\overline{W})\right] \overline{W}-3(1-\hat{\lambda }_{YW}^2)\hat{\gamma }(0),\\ c_3&=\left[ 2\hat{\lambda }_{YW}(\hat{\lambda }_{YW}+1)+ (1-\hat{\lambda }_{YW})(10-2(1-\hat{\lambda }_{YW})\overline{W})\right] \overline{W}-2(1-\hat{\lambda }_{YW}^2)\hat{\gamma }(0),\\ \mbox{and}~~~~~~~~~~~~~~~~~~~~~~~~~~~~~~~~~~~~~ c_4&=6(1-\hat{\lambda }_{YW})\overline{W}.\\ \end{aligned}$$

R packages for solving polynomial equations, particularly the cubic equation, abound. In “Yule–Walker approach” of this study, polyroot R function is used to obtain the roots of Eq. (23) after the coefficients have been calculated from the given time series. Though the equation has three roots, only the root that gives an acceptable value of $\hat{\delta }_{YW}$ will be used for further computations.

#### Approach to conditional least squares estimation

Consider the process in Eq. (12). Let $\hat{\varvec{\xi }}_{\text {CLS}}=\left( \hat{\lambda }_{CLS}, \hat{\delta }_{CLS} , \hat{\alpha }_{CLS}\right) ^T$ be the vector of the conditional least squares (CLS) estimators of ${\varvec{\xi }_{\text {CLS}}}=\left( {\lambda }_{CLS}, {\delta }_{CLS} , {\alpha }_{CLS}\right) ^T$. Then

$$\begin{aligned} \hat{\varvec{\xi }}_{\text {CLS}}=\underset{\varvec{\xi }}{\text {argmin}}(C_n(\varvec{\xi })), \end{aligned}$$

where $$C_n(\varvec{\xi })=\sum _{t=s+1}^n\left( W_t-\lambda W_{t-s}-(1-\delta ) \frac{\alpha +2}{\alpha (\alpha +1)}\right) ^2.$$

Here, it is necessary to obtain and equate each of $\frac{\partial C_n( \varvec{\xi })}{\partial \lambda } \big \vert _{\lambda =\hat{\lambda }_{CLS},~ \delta =\hat{\delta }_{CLS},~ \alpha =\hat{\alpha }_{CLS}},$,  

$\frac{\partial C_n( \varvec{\xi })}{\partial \delta } \big \vert _{\lambda =\hat{\lambda }_{CLS},~ \delta =\hat{\delta }_{CLS},~ \alpha =\hat{\alpha }_{CLS}}$ and $\frac{\partial C_n(\varvec{\xi })}{\partial \alpha }\big \vert _{\lambda =\hat{\lambda }_{CLS},~ \delta =\hat{\delta }_{CLS},~ \alpha =\hat{\alpha }_{CLS}}$ to zero, leading to the following equations, respectively:

25 $$\begin{aligned} \displaystyle \sum _{t=s+1}^nW_t W_{t-s}-\displaystyle \sum _{t=s+1}^n W_{t-s}^2-(1-\hat{\delta }_{\text {CLS}}) \frac{\hat{\alpha }_{\text {CLS}}+2}{\hat{\alpha }_{\text {CLS}} (\hat{\alpha }_{\text {CLS}}+1)}\displaystyle \sum _{t=s+1}^n W_{t-s}&=0; \end{aligned}$$

26 $$\begin{aligned} \displaystyle \sum _{t=s+1}^nW_t -\hat{\lambda }_{\text {CLS}} \displaystyle \sum _{t=s+1}^n W_{t-s}-(n-s)(1-\hat{\delta }_{\text {CLS}}) \frac{\hat{\alpha }_{\text {CLS}}+2}{\hat{\alpha }_{\text {CLS}} (\hat{\alpha }_{\text {CLS}}+1)}&=0; \end{aligned}$$

27 $$\begin{aligned} \displaystyle \sum _{t=s+1}^nW_t -\hat{\lambda }_{\text {CLS}} \displaystyle \sum _{t=s+1}^n W_{t-s}-(n-s)(1-\hat{\delta }_{\text {CLS}}) \frac{\hat{\alpha }_{\text {CLS}}+2}{\hat{\alpha }_{\text {CLS}} (\hat{\alpha }_{\text {CLS}}+1)}&=0. \end{aligned}$$

Equations (26) and (27) are identical. Hence, we solve for $\hat{\lambda }_{\text {CLS}}$ using Eqs. (25) and (26). The concerned estimator has the form:

28 $$\begin{aligned} \hat{\lambda }_{\text {CLS}}=\frac{(n-s)\sum _{t=s+1}^nW_t W_{t-s}-\sum _{t=s+1}^nW_t \sum _{t=s+1}^n W_{t-s}}{(n-s)\sum _{t=s+1}^n W_{t-s}^2-\left( \sum _{t=s+1}^n W_{t-s}\right) ^2}. \end{aligned}$$

Suppose $C_n(\delta , \alpha )$ is a function obtained when we substitute $\hat{\lambda }_{\text {CLS}}$ for ${\lambda }$ in $C_n(\varvec{\xi })$. We minimize $C_n(\delta , \alpha )$ in order to find $\hat{\delta }_{\text {CLS}}$ and $\hat{\alpha }_{\text {CLS}}$. The minimization process is done using a suitable numerical method. To summarize, no closed form expression can be found for any of $\hat{\delta }_{\text {CLS}}$ and $\hat{\alpha }_{\text {CLS}}$.

#### Conditional maximum likelihood estimation

In view of the s-step Markovian property of the seasonal INAR(1)$_s$ZMPL process, we define the transition probabilities as

$$\begin{aligned} P_{xy}&=P\left( X_t=y \big \vert X_{t-s}=x \right) \\&=P\left( \lambda *X_{t-s}+\nu _t=y \big \vert X_{t-s}=x \right) \\&=\sum _{r=0}^{y}P(\lambda *x=r)P(\nu _t=y-r).\\ \end{aligned}$$

Using this we can write the detailed transition probabilities as

29 $$\begin{aligned} P_{xy}=\left\{ \begin{array}{ll} \displaystyle \delta +(1-\delta )\frac{\alpha ^2(\alpha +2)}{(\alpha +1)^3}, &{}\text{ if }~~~x=0,~~y=0,\\ \displaystyle (1-\delta )\frac{\alpha ^2(y+\alpha +2)}{(\alpha +1)^{y+3}},&{}\text{ if }~~~x=0,~~y\ge 1, \\ \displaystyle \frac{1}{(1+\lambda )^x}\bigg (\delta +(1-\delta )\frac{\alpha ^2(\alpha +2)}{(\alpha +1)^3} \bigg ),&{}\text{ if }~~~x\ge 1,~~y=0, \\ \displaystyle \left( {\begin{array}{c}x+y-1\\ y\end{array}}\right) \frac{\lambda ^y}{(1+\lambda )^{x+y}}\bigg (\delta +(1-\delta )\frac{\alpha ^2(\alpha +2)}{(\alpha +1)^3}\bigg )\\ \displaystyle + \sum _{r=0}^{y-1} \left( {\begin{array}{c}x+r-1\\ r\end{array}}\right) \frac{\lambda ^r}{(1+\lambda )^{x+r}}\\ \displaystyle \times \bigg (\delta +(1-\delta )\frac{\alpha ^2(y-r+\alpha +2)}{(\alpha +1)^{(y-r+3)}} \bigg ) &{}\text{ if }~~~x\ge 1,~~y\ge 1. \end{array} \right. \end{aligned}$$

Let $\hat{\varvec{\xi }}_{\text {CML}}$ be the conditional maximum likelihood(CML) estimator of ${\varvec{\xi }}$. To find $\hat{\varvec{\xi }}_{\text {CML}}$, maximize the conditional log-likelihood function below:

$$\begin{aligned} l(\varvec{\xi })=\displaystyle \sum _{t=s+1}^n \text {log} P\left( X_t=x_t \big \vert X_{t-s}=x_{t-s}\right) . \end{aligned}$$

Obviously, $\hat{\varvec{\xi }}_{\text {CML}}$ has no closed form. Furthermore, the required estimators can only be found through a numerical technique.

### Simulation study for the INAR$(1)_{s}$ZMPL

In this section, we have carried out a simulation study to assess the performance of the proposed estimation methods for INAR$(1)_{s}$ZMPL process, paying attention to the cases of zero-inflation, overdispersion, zero-deflation and underdispersion baesd on the INAR$(1)_{s}$ZMPL process. We have simulated 1000 series of the sample sizes 100, 300, 500 and 1000 for various parameter combinations and for seasonal period $s=52$. We have computed the mean and the mean squared errors (MSEs) of the estimates over all the 1000 simulations. All the quantities are given in the Table 2, the first row for each parameter and sample size (*n*) combination represents the mean estimate and second row represents the MSE. From the table, it can be seen that the MSE is decreasing with the increase in sample size, indicating the consistency of the estimates. The numerical optimization of the likelihood function to obtain CML estimates is done using the function ‘*constrOptim*’ in R software. In our setup, constraints are imposed on all the parameters. In particular, the upper limit of $\delta$ is 1 while its lower limit is a negative quantity that is a function of $\alpha$. These constraints are considered while numerical optimization using ‘*constrOptim*’. In all, CML estimates correspond to minimum MSEs compared to the YW and CLS estimates.

**Table 2:** Parameter estimates and their mean squared errors.

*n*	$\hat{\lambda }_{ YW}$	$\hat{\alpha }_{ YW}$	$\hat{\delta }_{{ YW}}$	$\hat{\lambda }_{{ CLS}}$	$\hat{\alpha }_{{ CLS}}$	$\hat{\delta }_{ CLS}$	$\hat{\lambda }_{{ CML}}$	$\hat{\alpha }_{{ CML}}$	$\hat{\delta }_{{ CML}}$
$\lambda =0.3$, $\alpha =0.5$, $\delta =0.7$									
100	0.2739	0.6347	0.6307	0.2747	0.5749	0.6324	0.2952	0.5234	0.6919
	0.0157	0.0799	0.0205	0.0133	0.0159	0.0259	0.0081	0.0164	0.0054
300	0.2954	0.5363	0.6827	0.2947	0.5504	0.6641	0.2963	0.5073	0.6956
	0.0046	0.0083	0.0042	0.0046	0.0072	0.0077	0.0022	0.0036	0.0014
500	0.2953	0.5229	0.6868	0.2954	0.5396	0.6721	0.2993	0.5088	0.6963
	0.0029	0.0043	0.0026	0.0026	0.0046	0.0038	0.0013	0.0022	0.0009
1000	0.2966	0.5142	0.6919	0.2970	0.5303	0.6785	0.3005	0.5025	0.6998
	0.0013	0.0021	0.0012	0.0012	0.0029	0.0019	0.0006	0.0011	0.0004
$\lambda =0.5$, $\alpha =1$, $\delta =0.5$									
100	0.4319	1.1772	0.3866	0.4656	1.0993	0.4106	0.4838	1.0845	0.4713
	0.0198	0.4565	0.0848	0.0177	0.0358	0.0545	0.0138	0.0986	0.0190
300	0.4756	1.0852	0.4525	0.4864	1.0718	0.4472	0.4912	1.0121	0.4920
	0.0054	0.0560	0.0168	0.0050	0.0160	0.0147	0.0040	0.0209	0.0054
500	0.4865	1.0564	0.4689	0.4905	1.0668	0.4505	0.4954	1.0179	0.4905
	0.0032	0.0282	0.0097	0.0030	0.0114	0.0099	0.0022	0.0115	0.0028
1000	0.4928	1.0226	0.4867	0.4930	1.0622	0.4576	0.4988	1.0087	0.4973
	0.0015	0.0116	0.0043	0.0014	0.0089	0.0058	0.0011	0.0062	0.0014
$\lambda =0.6$, $\alpha =1.5$, $\delta =-0.5$									
100	0.5188	1.4169	$-0.4893$	0.5621	1.4184	$-0.4468$	0.5688	1.5475	$-0.5601$
	0.0172	0.9482	0.5433	0.0150	0.0833	0.0372	0.0124	0.1645	0.0796
300	0.5784	1.5685	$-0.5892$	0.5838	1.4341	$-0.4443$	0.5919	1.5326	$-0.5312$
	0.0043	0.4783	0.2972	0.0039	0.0382	0.0268	0.0028	0.0712	0.0354
500	0.5851	1.5689	$-0.5748$	0.5917	1.4460	$-0.4464$	0.5943	1.5242	$-0.5266$
	0.0026	0.2143	0.1535	0.0023	0.0261	0.0188	0.0017	0.0478	0.0239
1000	0.5936	1.5606	$-0.5558$	0.5961	1.4491	$-0.4494$	0.5971	1.5090	$-0.5107$
	0.0011	0.0649	0.0558	0.0011	0.0130	0.0130	0.0010	0.0225	0.0109
$\lambda =0.7$, $\alpha =2$, $\delta =-1$									
100	0.5804	1.4330	$-0.5686$	0.6590	1.8734	$-0.9271$	0.6653	1.9439	$-0.9821$
	0.0240	1.8765	1.2004	0.0153	0.1675	0.0744	0.0122	0.3010	0.1417
300	0.6713	1.7131	$-0.8222$	0.6858	1.8965	$-0.9120$	0.6891	2.0113	$-1.0204$
	0.0045	1.7318	1.0939	0.0038	0.0983	0.0559	0.0025	0.1510	0.0841
500	0.6848	1.8997	$-0.9649$	0.6906	1.9002	$-0.9101$	0.6936	2.0205	$-1.0274$
	0.0022	1.2074	0.8342	0.0022	0.0732	0.0491	0.0015	0.1214	0.0667
1000	0.6930	2.0364	$-1.0658$	0.6932	1.9088	$-0.9172$	0.6982	2.0339	$-1.0286$
	0.0011	0.7614	0.5599	0.0010	0.0393	0.0289	0.0007	0.0678	0.0378

### Model-based forecasting

Time series models are usually constructed with a view to forecasting future values of time series data. In the case of INAR$(1)_{s}ZMPL$ process, we handle the problem of forecasting a future observation $W_{n+h}, h \in \mathbb {N}$ using the information $\mathscr {F}_n$ available upto time *n*. From Eq. (12), we deduce using induction and properties of the NBT that

30 $$\begin{aligned} W_{n+h} \overset{\text {d}}{=} \lambda ^q*W_{n+h-qs}+\displaystyle \sum _{j=0}^{q-1} \lambda ^q* \nu _{n+h-js}, \quad h\in \mathbb {N}^+, \end{aligned}$$

where $\overset{\text {d}}{=}$ implies equality in distribution, $q=\lceil \frac{h}{s}\rceil$ is the integer part of   $\frac{h}{s}$. That is $\lceil y \rceil =\text {min}\left[ n \in \mathbb {N}^+ \big \vert y \le n \right]$. We round off this section with the following proposition.

#### Proposition 3.4

*Consider the INAR(1)*$_s$ *ZMPL process in Eq*. (12). *Then*

- *The h-step conditional expectation is*
  $$\begin{aligned} \text {E}\left( W_{n+h} \big \vert \mathscr {F}_n\right) = \lambda ^q \left( W_{n+h-qs}-\text {E}\left( W_t\right) \right) +\mu _\nu . \end{aligned}$$
- *The h-step conditional variance is*
  $$\begin{aligned} \text {Var}\left( W_{n+h} \big \vert \mathscr {F}_n\right)&= (1+\lambda )\frac{\lambda ^{q}\left( 1-\lambda ^{q}\right) }{1-\lambda }W_{n+h-qs} \\ {}&\quad +\frac{\lambda (1+\lambda )}{1-\lambda } \left( \frac{1-\lambda ^{2(q-1)}}{1-\lambda ^{2}}-\frac{\lambda ^{(q-1)}\left( 1-\lambda ^{(q-1)}\right) }{1-\lambda }\right) \mu _\nu \\ {}&\quad +\left( \frac{1-\lambda ^{2q}}{1-\lambda ^2}\right) \sigma _\nu ^2. \end{aligned}$$
- $$\lim \nolimits _{q \rightarrow \infty } \text {E}\left( W_{n+h} \big \vert \mathscr {F}_n\right) = \text {E}\left( W_t \right) .$$
- $$\lim \nolimits _{q \rightarrow \infty } \text {Var}\left( W_{n+h} \big \vert \mathscr {F}_n\right) = \text {Var}\left( W_t \right) .$$

It is not difficult to prove Proposition 3.4 using knowledge of the proofs of Propositions 3.1 to 3.2 and Eq. (30). Therefore, we omit the proof of Proposition 3.4. The *h*-step ahead forecast can be written as

$$\begin{aligned} \hat{W}_{n+h}=\hat{\lambda }^q\left( \hat{W}_{n+h-qs}- \hat{\mu }_W\right) +\hat{\mu }_\nu . \end{aligned}$$

### Model comparison with seasonal ARIMA

In this section, we have compared the forecast performance of the proposed model and its counterpart $SARIMA(0,0,0)(1,0,0)_{s}$ model. Here, we have simulated 1000 series each of size 500 from the INAR(1)$_s$ ZMPL model with various parameter combinations given in Table 3 and the seasonal period $s=52$. For each series, the first 495 observations are used for the estimation of the parameters and last five observations are used to check the forecast accuracy. We have used three forecast accuracy measures to assess the forecast performance of the model. These measures include prediction root mean squared error (PRMSE), prediction mean absolute error (PMAE) and percentage of true prediction (PTP). Notably,

$$\begin{aligned} PRMSE(k)= \sqrt{\frac{1}{m}\sum \limits _{i=1}^{m}\left( X_{(t+i)}-\hat{X}_{(t+i)}^{(k)}\right) ^2}, \end{aligned}$$

where $\hat{X}_{(t+i)}^{(k)}=\widehat{mean}(X_{t+i}|X_{t-k+i})$ is the *k*-step ahead conditional mean of the fitted process,

$$\begin{aligned} PMAE(k)=\frac{1}{m}\sum \limits _{i=1}^{m}| X_{(t+i)}-\hat{X}_{(t+i)}^{(k)}|, \end{aligned}$$

where $\hat{X}_{(t+i)}^{(k)}=\widehat{median}(X_{t+i}|X_{t-k+i})$ is the *k*-step ahead conditional median of the fitted process, and

$$\begin{aligned} PTP(k)=\frac{1}{m}\sum \limits _{i=1}^{m}I\Big (X_{(t+i)}=\hat{X}_{(t+i)}^{(k)}\Big )\times 100\%, \end{aligned}$$

where $I(\cdot )$ denotes the indicator function. Here, $X_{(t+i)}$ is the actual *i*th observation at time point $(t + i)$ and $\hat{X}_{(t+i)}^{(k)}$ is the *k*-step ahead forecast value at the same time point.

We have obtained the conditional mean of the process as traditional forecast while the median and mode from one-step ahead forecast distribution serve as coherent forecasts. For the computation of PMAE in the SARIMA model, we have used mean forecast while in the INAR(1)$_s$ ZMPL model, we have used the median of the one-step ahead forecast distribution. In the SARIMA model, we can have only mean as the forecast, for computation of percentage of true prediction (PTP) based on mean we have considered rounded mean for both models. We have computed median and mode from the forecast distribution for the computation of PTP-Med and PTP-Mode. In INAR literature, the median and mode of the forecast distribution are called the coherent forecasts.

From Table 3, it can be seen that in terms of PRMSE both models perform equally well, as their forecasts are the same. However, the PMAE for the SARIMA model is higher than that of the PMAE for the proposed model, showing the better performance of the proposed model than the SARIMA model. This may be because median is used in the proposed model and mean is used in the SARIMA model for the computation of the PMAE. But when it comes to the coherent forecast performance, the proposed model outperforms the SARIMA model, which can be seen from the PTP for median and mode. When data are overdispersed, as it is the case in the top panel in the table, the coherent forecast performs five times better than the traditional forecast. Both models perform equally well when the data are underdispersed.

**Table 3:** Forecast comparison with SARIMA.

	SARIMA			INAR(1)$_s$ ZMPL				
k	PRMSE	PMAE	PTP	PRMSE	PMAE	PTP-Mean	PTP-Med	PTP-Mode
$$\lambda =0.3,~\alpha =0.5,~\delta =0.7$$								
1	2.376	1.555	10.00	2.374	1.186	9.80	58.10	61.30
2	2.622	1.727	8.90	2.618	1.411	9.50	55.00	57.30
3	2.602	1.679	8.10	2.592	1.300	8.20	59.20	60.20
4	2.545	1.643	9.10	2.546	1.244	8.50	58.60	62.80
5	2.443	1.595	10.20	2.438	1.203	9.80	60.30	62.30
$$\lambda = 0.5,~\alpha =1,~\delta =0.5$$								
1	1.787	1.310	17.50	1.784	1.140	17.50	43.10	47.50
2	1.671	1.254	16.60	1.673	1.035	16.90	45.20	51.10
3	1.791	1.322	16.10	1.788	1.122	16.00	43.30	50.60
4	1.809	1.335	15.30	1.810	1.134	15.00	42.30	49.50
5	1.834	1.335	17.00	1.832	1.128	17.00	46.00	48.70
$$\lambda = 0.6,~\alpha =1.5,~\delta =-0.5$$								
1	2.391	1.730	19.60	2.391	1.701	19.60	22.20	23.70
2	2.209	1.677	19.00	2.213	1.645	18.70	20.70	22.20
3	2.483	1.868	15.60	2.470	1.839	15.10	18.00	21.60
4	2.307	1.705	19.50	2.302	1.674	18.90	20.80	22.00
5	2.300	1.719	19.50	2.294	1.683	19.20	20.90	22.70
$$\lambda = 0.7,~\alpha =2,~\delta =-1$$								
1	2.584	1.875	19.20	2.587	1.858	18.90	19.90	20.30
2	2.448	1.856	17.30	2.442	1.841	17.30	18.10	20.30
3	2.586	1.873	18.90	2.575	1.849	18.50	20.50	22.00
4	2.565	1.862	20.90	2.562	1.846	20.30	21.10	19.80
5	2.752	1.959	21.30	2.736	1.953	21.00	21.60	20.20

### Application of INAR(1)$_s$ZMPL model

**Figure 2:**
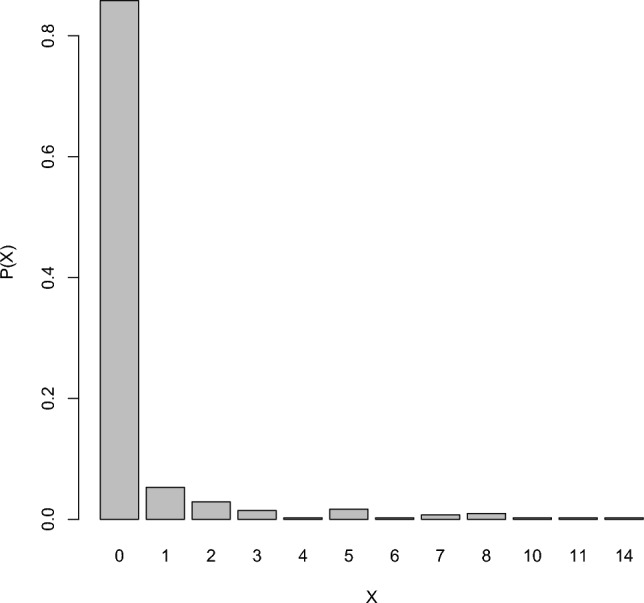
Relative frequency plot for the influenza data.

**Figure 3:**
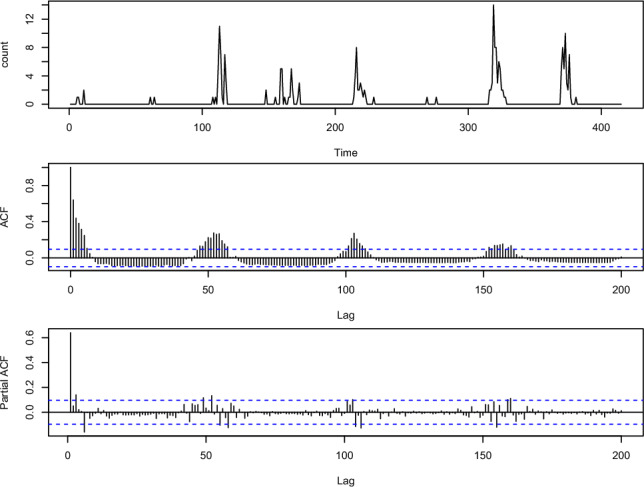
Sample path and sample ACF influenza data.

**Figure 4:**
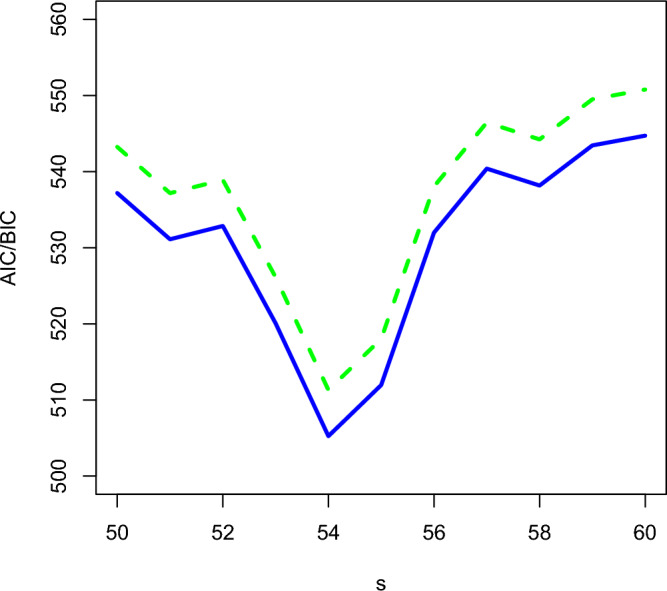
Akaike information criterion (AIC) and Bayesian information criterion (BIC) for influenza data for various seasonal periods ‘*s*’. Green dashed line is for the BIC and Blue solid line is for AIC.

We have analyzed weekly Influenza data from the Breisgau- Hochschwarzwald county of Baden–Württemberg state, Germany for the years 2001 to 2008 (https://survstat.rki.de). The data have mean 0.4735 and variance 2.5969. Clearly the data are overdispersed. From the relative frequency plot in Fig. 2, we can see that the number of zeros in the data is excessively high. Hence, this series can be modeled using the proposed zero-modified model. The seasonality of the data can be seen from the ACF and PACF plot in Fig. 3, as it is characterized by the sinusoidal autocorrelation pattern. Also, the data are seasonal because the corresponding ACF plot has significant peaks at multiples of 54. From Fig. 4, it can be seen that the AIC and BIC values are small for the period $s=54$. Hence the seasonal period of the series ($s=54$) is confirmed.

**Table 4:** Model selection among seasonal INAR models using AIC and BIC.

Model	Parameter estimates	AIC	BIC
PINAR(1)$_S$^14^	$\hat{\phi }$=0.2435, $\hat{\lambda }$=0.4288	939.950	948.011
GINAR(1)$_{S}$^13^	$\hat{\phi }$=0.4891, $\hat{\theta }$=0.4993	627.946	636.946
SNGINAR(s)^16^	$\hat{\alpha }$=0.2900, $\hat{\mu }$=0.6499	660.493	668.554
INAR(1)$_s$ZMPL	$\hat{\lambda }=0.2509$, $\hat{\alpha }=0.5286$, $\hat{\delta }$=0.8640	505.243	517.535

From Table 4, it can be seen that the proposed model is the best model for the data. We have obtained point forecast using the INAR(1)$_s$ZMPL model for the given data. The data has 416 observations. We have used 411 observations (training data) for estimating the parameters and last five observations are predicted using the fitted model. We have used the conditional ‘*h*’ step ahead mean as the forecast function. The parameter estimates (CML) based on training data are $\hat{\lambda }=0.2497$, $\hat{\alpha }=0.5289$, $\hat{\delta }=0.8619$. The mean forecast $\hat{X}_{t+h}$ and the rounded mean forecast [$\hat{X}_{t+h}$] are given in the Table 5. From this table it can be seen that the model predicts the future observations with good accuracy.

**Table 5:** Point forecasts for the influenza data.

*h*	$X_{411+h}$	$\hat{X}_{411+h}$	[$\hat{X}_{411+h}$]
1	0	0.2850	0
2	0	0.2850	0
3	0	0.2850	0
4	0	0.2850	0
5	0	0.2850	0

## Conclusion

In this paper, we have introduced a seasonal, nonnegative, integer-valued autoregressive model, with multiple features, using the negative binomial thinning operator and zero-modified Poisson-Lindley distributed innovations. General seasonal INAR model of order *P* has been discussed in the paper and it has been illustrated in detail for order one. This model, denoted by INAR(1)$_s$ZMPL model, is the first of its kind for modeling zero-deflated or zero-inflated seasonal time series of counts. Some theoretical results are determined for the model. Specifically, means, variances and autocorrelation function are obtained. In estimating the parameters of the model, the Yule-Walker, conditional least squares and conditional maximum likelihood approaches are given due consideration. The simulation results obtained in this study demonstrate the superiority of the conditional maximum likelihood method over the other two point estimation methods of estimating the model parameters. A real-life application of the model was investigated by analyzing a zero-inflated overdispersed seasonal count time series. The model fit was compared to the fits of three competing models. In the final analysis, the proposed model outperforms the other models.

## Data Availability

The datasets used and/or analysed during the current study can be provided by the corresponding author upon reasonable request.
